# A case of endoscopic ultrasound-guided vascular intervention for pancreaticojejunal varices via a gastric approach

**DOI:** 10.1055/a-2791-4604

**Published:** 2026-02-13

**Authors:** Tomoki Ogata, Kazuo Hara, Nozomi Okuno, Shin Haba, Takamichi Kuwahara, Shimpei Matsumoto, Hiroki Koda

**Affiliations:** 1 538357 Department of Gastroenterology, Aichi Cancer Center Hospital, Nagoya, Japan

Recurrent pancreatic cancer can obstruct the portal venous flow, leading to collateral formation and variceal bleeding. Anastomotic variceal bleeding following pancreaticoduodenectomy is extremely rare and difficult to control using standard endoscopic techniques 1 . We have previously reported the usefulness of endoscopic ultrasound (EUS)-guided embolization of varices around the pancreaticojejunostomy via anastomosis 2 . Nonetheless, EUS-guided transgastric embolization of intraperitoneal varices has not yet been reported.

A woman in her 30s underwent pancreaticoduodenectomy for pancreatic head cancer, performed at X-4 years. Recurrence was observed after X-3 years. In October X-1, the patient developed progressive anemia and melena.

**Fig. 1:**
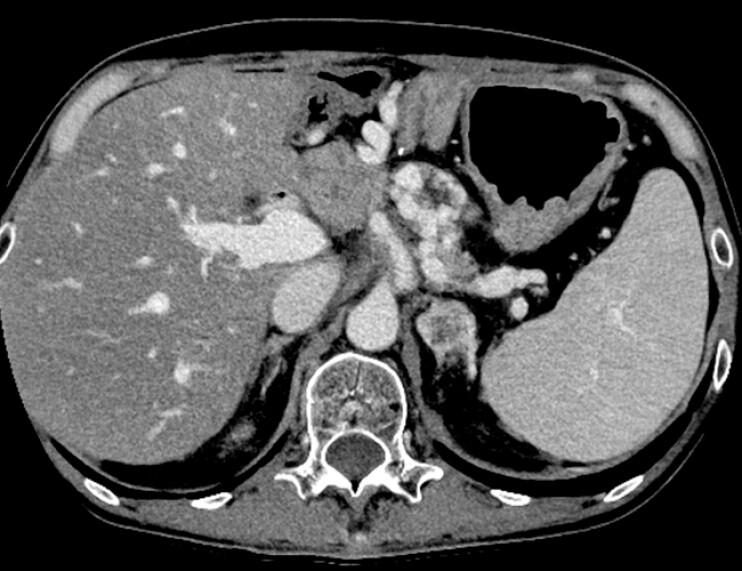
CT revealed occlusion of the splenic vein due to recurrent pancreatic head cancer, with the development of varices around the pancreaticojejunostomy. CT, computed tomography.

Contrast-enhanced computed tomography (CT) revealed splenic vein occlusion due to cancer recurrence with the development of varices around the pancreaticojejunostomy site, which were suspected to be the cause of progressive anemia and melena ( Fig. 1 ).

**Fig. 2:**
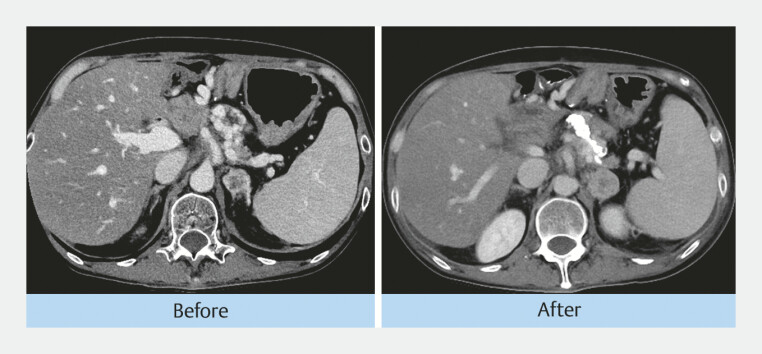
This is a CT image before and after EUS-guided embolization, showing embolic material within the varices. CT, computed tomography; EUS, endoscopic ultrasound.

**Fig. 3:**
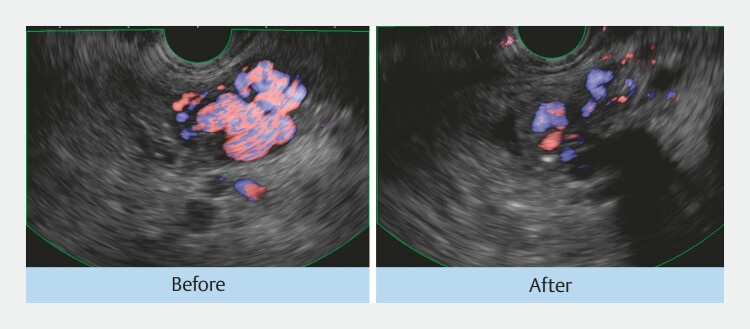
This is the EUS image before and after the embolization, showing a reduction in the color doppler signal within the varices. EUS, endoscopic ultrasound.

Direct endoscopic access to the anastomosis was not possible due to dense adhesions. Therefore, we attempted to perform EUS-guided transluminal embolization of intraperitoneal varices. Varices around the pancreaticojejunostomy site were identified using color Doppler imaging with an oblique-viewing echoendoscope (EG-740UT; FUJIFILM Medical, Tokyo, Japan); subsequently, they were punctured with a 22-gauge needle (EZ Shot 3 Plus; Olympus, Tokyo, Japan), followed by the injection of a mixture of n-butyl-2-cyanoacrylate and ethiodized oil (1.5 mL of n-butyl-2-cyanoacrylate and 0.5 mL of ethiodized oil; Video 1 ). Post-procedure imaging confirmed effective embolization: CT demonstrated an embolic material within the varices, and EUS identified a color Doppler signal reduction ( Fig. 2 , Fig. 3 ). The patient experienced no adverse events and was discharged on postoperative day 5. She remained free from anemia or rebleeding for 6 months until death from the primary disease.


A visualized splenic vein. Visualized varices from the splenic vein. Varices were visualized along their longest axis. Puncture of the varices with 22G EZ Shot 3 Plus. Saline was injected to confirm the correct puncture. A mixture of *N* -butyl-2-cyanoacrylate and ethiodized oil was injected into the varices. The color signal was decreased.
Video 1

This case demonstrates that, even in patients with adhesions after pancreaticoduodenectomy, where access from the anastomotic side is difficult, a transgastric EUS-guided approach can serve as a safe and effective therapeutic option.

Endoscopy_UCTN_Code_TTT_1AS_2AL Endoscopy_UCTN_Code_CCL_1AF_2AZ_3AD
